# Tailored conditioning for hematopoietic stem cell transplantation in children with Mendelian susceptibility to mycobacterial disease: A tale of two pathways for IFN-γ and IL-12 defects

**DOI:** 10.70962/jhi.20250264

**Published:** 2026-07-27

**Authors:** Kavitha Ganesan, Minakshi Balwani, Nithya Seshadri, Vijayashree Muthukumar, Krithika Krishnakumar, Anuraag Nalla Reddy, Vidya Krishna, Indira Jayakumar, Ramya Uppuluri, Revathi Raj

**Affiliations:** 1 Department of Pediatric Hematology-Oncology, Blood and Marrow Transplantation Unit, Apollo Hospitals, Chennai, India; 2 Institute of Infectious Disease, Apollo Hospitals, Chennai, India; 3 Department of Pediatric Intensive Care Unit, Apollo Hospitals, Chennai, India

## Abstract

Mendelian susceptibility to mycobacterial disease (MSMD) is a rare group of inborn errors of immunity associated with significant morbidity due to mycobacterial infections. Hematopoietic stem cell transplantation (HSCT) is a curative option; however, persistent infections and high levels of interferon-γ (IFN-γ) create a hostile environment for stem cell proliferation and can mediate graft rejection. We describe the outcome of children with MSMD due to IFN-γ receptor and interleukin-12 (IL-12) defects who underwent HSCT in our unit from December 2015 to December 2023. We collected the data retrospectively from a review of patient charts and analyzed the outcomes. Seven children underwent eight procedures, including four with IFN-γ defects and three with IL-12 defects. The male-to-female ratio was 1.6:1, and the median age at HSCT was 4.5 years. Five received haploidentical HSCT, and two underwent matched family donor transplants; 75% used peripheral blood stem cells. IL-12–deficient cases engrafted well with fludarabine/treosulfan–based conditioning, whereas IFN-γ–deficient cases fared better with fludarabine/treosulfan/thiotepa-based conditioning due to a higher rejection risk. Acute graft-versus-host disease (GVHD) (grade 1–2, skin/gut) occurred in two children: one developed chronic liver GVHD after donor lymphocyte infusion. Cytomegalovirus reactivation occurred in four cases, which responded to ganciclovir or cidofovir. One primary graft failure was rescued with a second HSCT; one secondary graft failure led to death from invasive fungal infection. At a median follow-up of 8 years, the overall survival was 87%. One survivor has ongoing seizures, aggressive behavior, and endocrine dysfunctions requiring supportive care. HSCT is a curative option for children with MSMD. However, we need to understand gene mutation and individualize the approach to preparation and conditioning regimens to help optimize the outcome.

## Teaser

Hematopoietic stem cell transplantation is a curative option for children with MSMD. Outcomes improve when conditioning is tailored to genetic defects: Flu/Treo for IL-12 deficiency and Flu/Treo/TT with pretransplant immunosuppression for IFN-γ defects. Multidisciplinary infection management and long-term follow-up are essential to optimize survival and quality of life.

## Introduction

Mendelian susceptibility to mycobacterial disease (MSMD) is a rare group of inborn errors of immunity characterized by a selective predisposition to mycobacterial infections, including bacillus Calmette–Guérin (BCG)–derived *Mycobacterium bovis* and environmental mycobacteria (1, 2). Although the condition typically manifests in childhood, adult-onset cases have also been reported (3). In countries where BCG vaccination is routinely administered at birth, some affected newborns may first present with complications following vaccination, particularly in a developing country like India (4). Since BCG is contraindicated in patients with MSMD and their newborn siblings until genetic confirmation, most diagnoses are made only after adverse events occur after vaccination. However, delaying or suspending BCG vaccination may pose significant public health challenges in tuberculosis-endemic areas such as India, where the vaccine remains a critical preventive measure, where newborn screening tests are not available, and delaying vaccination can be more harmful.

The interleukin-12 (IL-12)/IL-23/ISG15–interferon-γ (IFN-γ) axis plays a pivotal role in host defense against mycobacterial infections. Defects in any component of this pathway impair IFN-γ production and/or signaling, leading to MSMD. Such abnormalities predispose affected individuals to severe, disseminated, or recurrent infections caused by environmental mycobacteria, *M*.* bovis*, BCG from vaccination, *Salmonella* non-typhi, and other intracellular pathogens. According to the International Union of Immunological Societies, pathogenic variants in an increasing number of genes are implicated in MSMD, including autosomal genes (*IL12RB1*, *IL12B*, *IL12RB2*, *IL23R*, *IFNGR1*, *IFNGR2*, *STAT1*, *IRF8*, *SPPL2A*, *TYK2*, *ISG15*, *RORC*, and *JAK1*) and the X-linked gene (*CYBB*) (5, 6).

These genetic mutations primarily affect the IFN-γ/IL-12/IL-23 signaling pathway, thereby compromising the immune response to mycobacteria (3, 7). This immunodeficiency affects the innate and adaptive immunity, leading to a range of clinical manifestations (5, 8). The diagnosis of MSMD requires a comprehensive approach that integrates clinical assessment, microbiologic testing, immunologic evaluation, and genetic sequencing (9).

Treatment and management of MSMD requires individualized strategies tailored to the underlying genetic defect. These typically include antimicrobial therapy, IFN-γ replacement, hematopoietic stem cell transplantation (HSCT), and, more recently, gene therapy. While prolonged antimycobacterial treatment remains the cornerstone of care, IFN-γ therapy can be beneficial in select patients. However, HSCT may remain the only curative option in this condition, and there are only a handful of case series reported worldwide. Hence, this case series underscores the challenges encountered during HSCT in children with MSMD and outlines practical solutions developed to overcome them.

## Results

A total of seven children underwent eight HSCTs at our center for underlying MSMD involving the IL-12 and IFN-γ pathways. The cohort included four children (57%) with confirmed genetic mutations affecting the IFN-γ pathway and three children (43%) with an IL-12 pathway defect. Table 1 describes the patient, donor, and treatment details. The male-to-female ratio was 1.6:1 (male: 62.5%, female: 37.5%), and the median age at transplantation was 4.5 years (range: 6 mo to 10 years).

**Table 1.** Patient characteristics and outcomes

Patient	Age/Sex	​	Donor type	Conditioning regimen	Stem cell (CD34 cells) dose per kg of recipient	Post-HSCT outcome
1	3 mo/male	IFN-γ	Haplo	Flu/Treo/PTCy	5 million	Graft rejection/alive
2	5 years/male	IFN-γ	MFD	Flu/Treo/TT	5 million	Graft rejection/dead
3	3 years/female	IFN-γ	MSD	Flu/Treo/TT	10 million	Complete donor chimerism/alive
4	2.5 years/female	IFN-γ	Haplo	Flu/Treo/TT TCRαβ depletion	10 million	Complete donor chimerism/alive
1	6 years/male (second HSCT)	IFN-γ	Haplo	Flu/Treo/TT TCRαβ depletion	10 million	Mixed chimerism—improved after graded whole blood DLI/alive
5	5 years/male	IL-12	MFD	Flu/Treo	7 million	Complete donor chimerism/alive
6	10 years/male	IL-12	Haplo	Flu/Treo/2 Gy TBI/PTCy	8 million	Complete donor chimerism/alive
7	6 years/female	IL-12	Haplo	Flu/Treo/PTCy	8 million	Mixed chimerism—improved after graded whole blood DLI/alive

MSD, matched sibling donor; TBI, total body irradiation.

Five children (62.5%) received haploidentical HSCT, while three (37.5%) underwent matched family donor (MFD) transplants. Of the children who underwent haploidentical HSCT, three had T cell–replete transplantation, while the remaining two had TCRαβ depletion/CD19 depletion transplantation. Peripheral blood stem cells were used as the graft source in six transplants (75%), while bone marrow was used in two transplants (25%).

Conditioning regimens varied according to the underlying genetic defect. Patients with IL-12 pathway mutations (*n* = 3) received fludarabine/treosulfan (Flu/Treo) conditioning and demonstrated favorable engraftment kinetics without significant early transplant-related complications. In contrast, patients with IFN-γ pathway defects (*n* = 4) were administered fludarabine/treosulfan/thiotepa (Flu/Treo/TT) after the experience of patient 1, who had a primary graft rejection with Flu/Treo-based conditioning.

Engraftment was achieved in seven of eight transplants (87.5%). One child with an IFN-γ defect experienced primary graft failure (12.5%) but subsequently underwent a second HSCT as described above using a different donor and conditioning protocol, resulting in successful long-term engraftment. Another child (patient 2) developed secondary graft failure 3 mo after transplant and succumbed to complications from invasive fungal infection, resulting in a transplant-related mortality rate of 12.5%.

Patient 1 initially underwent haploidentical HSCT from his father as donor with Flu/Treo-based conditioning. He had primary graft failure with autologous reconstitution and was restarted on antitubercular therapy (ATT). An attempt was made to stop ATT, after which he developed fever, skin rash, lymphadenopathy, and organomegaly, which responded to ATT. Following that, the child had multiple infective episodes, including documented *Salmonella* infection, an episode of hemophagocytic lymphohistiocytosis, and respiratory syncytial virus pneumonia requiring high-flow oxygen support. He developed hypersplenism with increasing requirement for blood transfusions, cytopenia, and splenomegaly, and had to undergo splenectomy. With increasing comorbidities, a second HSCT was indicated and was planned after discussions with the family regarding challenges and possible mortality. A novel strategy to decrease IFN-γ levels was employed, including two cycles of pretransplant immunosuppression (PTIS) with Flu/dexamethasone, double-volume plasma exchange, and rituximab. He underwent a TCRαβ-depleted haplo-HSCT with peripheral blood stem cells from his mother, with infusion of a stem cell dose of 10*10^6^ cells/kg CD34, and engrafted well. This strategy proved effective, with successful engraftment observed in three of four children in this group with IFN-γ defect.

Donor chimerism is routinely checked on days 15, 30, 45, 60, and 90, 6 mo, and 1 year after HSCT. For patient 1 with mixed chimerism, three whole blood donor lymphocyte infusions (DLIs) were performed at 2-weekly intervals at graded doses of 1*10^5^/kg, 5*10^5^, and 1*10^6^/kg. We documented serially increasing chimerism after the third DLI. For patient 7, one whole blood DLI was performed and subsequently had complete chimerism. Both the children were monitored for the presence of graft-versus-host disease (GVHD) during DLI.

Acute GVHD was observed in two children (25%), both presenting with grade I–II involvement of the skin and gastrointestinal tract. Chronic GVHD of the liver occurred in one child (patient 1) following DLI administered for mixed chimerism. This case was managed successfully with immunosuppressive therapy including steroids, mycophenolate mofetil, cyclosporine, ruxolitinib, and five sessions of extracorporeal photopheresis.

CMV reactivation occurred in four children (50%), with viral loads ranging up to 1.9 × 10^5^ copies/ml. All patients responded well to antiviral therapy with ganciclovir and cidofovir, and no CMV-related mortality was documented. One child (patient 7) developed CMV-induced nephropathy, which was managed with prolonged supportive care, including angiotensin-converting enzyme inhibitors and diuretic therapy with furosemide. Clinical and biochemical improvement was noted as the CMV viral load decreased and eventually achieved complete renal recovery. At the time of publication, the child continues to be well, with no evidence of long-term renal morbidity or functional impairment.

The overall survival in the cohort was 87.5% (7/8 transplants). All surviving children were on long-term follow-up ranging from 2 to 8 years after HSCT (median follow-up: 4.5 years). Long-term outcomes were favorable in the majority, with stable donor chimerism and immune reconstitution. Antitubercular medications were continued for about 18 mo after HSCT or after immune reconstitution in these children, whichever was earliest. On long-term follow-up, however, one child continued to experience significant neurobehavioral and endocrine sequelae, including the absence of seizures, aggression, and multiple hormone deficiencies requiring ongoing endocrinology and neurology care as a sequela to pretransplant neurotuberculosis. Table 1 demonstrates patient characteristics and outcomes.

## Discussion

Our findings highlight that HSCT can be a curative and feasible therapeutic option for patients with MSMD, even in the presence of disseminated mycobacterial infection or in the absence of a fully HLA-matched donor. The need for prolonged ATT until adequate immune reconstitution must be emphasized to the family prior to starting transplantation. Collaborative effort with an intensivist and an infectious specialist helps many of these children have a better quality of life after successful HSCT.

In India, mycobacterial tuberculosis continues to be a major public health challenge, with the incidence of drug-resistant strains escalating at a rapidly concerning pace. These resistant mycobacterial infections can have dreadful complications, particularly for children with immunodeficiency, and long-term care becomes difficult in resource-limited settings. The nonavailability of IFN-γ therapy in many developing countries leaves few treatment options behind. For now, HSCT remains the only long-term curative option available for children and families with MSMD (10, 11, 12).

Patient selection is the key and can be challenging. Most of the children present with disseminated tuberculosis in a sick state with multidrug-resistant infection and poor nutritional status. Bringing them into a clinically stable condition before giving high-dose chemotherapy is difficult, and many children experience increased toxicity during conditioning. Roesler et al. reported a multicenter survey in which two children with active mycobacterial infection died after HSCT and recommended optimal control of mycobacterial infection before HSCT and use of a non-T cell–depleted transplant from an HLA-identical sibling after a fully myeloablative conditioning regimen (13). Similar results have been reported by others that achieving disease remission before HSCT affects outcome and immune reconstitution (14, 15).

However, complete control of disease prior to HSCT is difficult as there may be severe intercurrent infections in these children, which can lead to mortality. Hence, with the infectious disease and pediatric intensive care unit team collaboration, these children remain on antitubercular medication prior to, during, and after the transplantation period to prevent flare-up of the disease. Local control of infection, if needed, is also done prior to HSCT.

Many previous studies have reported improved success rates with matched donors; however, with decreasing family size and reduced family donor availability, HSCT is not uniformly accessible to all children. Haploidentical HSCT is an attractive option as the donor is readily available for almost all patients, especially in children with a narrow stable window for proceeding with HSCT.

In the study by Rottman et al., the most significant finding was the high incidence of graft failure or declining donor chimerism (16). Patients with IL-12 defect tolerated reduced-intensity conditioning with fludarabine and treosulfan, while children with IFN-γ defect had high rates of graft rejection as seen in our series. Mouse model studies suggest that elevated pretransplant IFN-γ levels contribute to this rejection risk, as IFN-γ inhibits stem cell proliferation and hematopoiesis. Since recipient hematopoietic cells lack IFN-γ receptors, they gain a selective growth advantage, leading to autologous reconstitution (17) as was seen in the first child in the cohort. Due to the nonavailability of emapalumab, which is an IFN-γ receptor inhibitor used predominantly in children with primary hemophagocytic lymphohistiocytosis, protocol modification was done to lower the rate of IFN-γ using pretransplantation immunosuppression with Flu, dexamethasone, and plasma exchange (18).

Challenges in the cohort included viral reaction and mixed chimerism. Significant viral reactivation with end-organ damage in one child due to CMV was seen in the cohort, but all of them responded to antiviral agents. With the recent introduction of letermovir in the market, the incidence of significant viral reactivation rates is in a declining trend. The mixed chimerism required graded DLI, after which chimerism improved; however, the child developed chronic liver GVHD.

Limitations of the study include the retrospective study design, small number of patients, and lack of data on IFN-γ levels before and after intervention in these patients. We plan to conduct a national multicentric prospective trial to validate the results.

## Materials and methods

We describe retrospective outcome of children with MSMD due to IFN-γ receptor and IL-12 defects who underwent HSCT in our unit from December 2015 to December 2023 over an 8-year period. We collected the data retrospectively from a review of patient charts. The data collected included patient demographics, type of HSCT, donor type, source of stem cells used, conditioning regimen used, number of stem cells infused, engraftment details, incidence of viral reactivation, incidence of GVHD, and overall survival.

Prior to conditioning and starting the HSCT procedure, all children were screened for viruses in blood, stool, and respiratory samples. A whole-body positron emission tomography CT scan was performed to screen for any focus of infection, and a consultation from the infectious disease team was obtained before HSCT.

Children with IFN-γ defect received Flu (40 mg/m^2^/day for 4 days), Treo (14 g/m^2^/day for 3 days), and TT (5 mg per kg per dose two times a day for 1 day), while the children with IL-12 defect received Flu (40 mg/m^2^/day for 4 days) and Treo (14 g/m^2^/day for 3 days). The CliniMACS system was used for ex vivo T cell depletion, and posttransplant cyclophosphamide (PTCy) was used for the T cell–replete method.

In view of graft rejection in a child who received Flu/Treo for a haploidentical HSCT with IFN-γ defect (patient 1), PTIS with two cycles of Flu (40 mg/m^2^ for 5 days) and dexamethasone (25 mg/m^2^) followed by double-volume plasma exchange, Flu/Treo/TT conditioning, and a high stem cell dose of 10 million cells per kg of recipient was performed in children with IFN-γ receptor, while children with IL-12 defect received Flu/Treo conditioning regimen.

For GVHD prophylaxis, short-course methotrexate and a calcineurin inhibitor (tacrolimus) were used for the children undergoing MFD HSCT. In children receiving T cell–replete transplants, PTCy was used with tacrolimus, while T cell–depleted HSCT children received cyclosporine as an immunosuppressant. GVHD prophylaxis was continued for a minimum period of 1 year in MFD and 18 mo in haploidentical HSCT or until immune reconstitution, whichever was later.

The children were also on prophylaxis with cotrimoxazole for *Pneumocystis *pneumonia and itraconazole as antifungal prophylaxis prior to HSCT and received regular monthly intravenous immunoglobulin. ATT was administered prior to HSCT for disease control and after HSCT until immune reconstitution or at least 18 mo.

Viral monitoring through PCR technique for CMV, adenovirus, and Epstein-Barr virus was done once weekly until 100 days after HSCT, and preemptive treatment was initiated based on viral reactivation.

Donor chimerism is routinely checked on days 15, 30, 45, 60, and 90, 6 mo, and 1 year after HSCT. For patients with mixed chimerism, whole blood DLI was performed at 2-weekly intervals at graded doses. As we document serially increasing chimerism, DLI was stopped and monitored for the presence of GVHD in these children.

The study has been approved by the institutional review board, and written informed consent was obtained from parents/guardians of all children.

### Conclusion

MSMD can be associated with significant morbidity and mortality, and prolonged ATT can be challenging, particularly in low- and middle-income countries, where there is a high prevalence of tuberculosis among the population and drug-resistant tuberculosis predominates. HSCT can be potentially curative; however, it is fraught with challenges. PTIS with Flu and dexamethasone, followed by plasma exchange, use of Flu/Treo/TT, and providing a high stem cell dose, is needed for durable engraftment and to prevent graft rejection in children, especially with IFN-γ receptor defect. The success of HSCT in this challenging patient population is influenced by advances in conditioning regimens, optimized graft manipulation techniques, and the use of prolonged, targeted ATT.

## Data Availability

Data are available upon request.
